# A framework for continuous target tracking during MR-guided high intensity focused ultrasound thermal ablations in the abdomen

**DOI:** 10.1186/s40349-017-0106-y

**Published:** 2017-10-09

**Authors:** Cornel Zachiu, Baudouin Denis de Senneville, Ivan D. Dmitriev, Chrit T. W. Moonen, Mario Ries

**Affiliations:** 1 Imaging Division, UMC Utrecht, Heidelberglaan 100, Utrecht, 3508 GA Netherlands; 2 Institut de Mathématiques de Bordeaux, CNRS UMR5251/Université de Bordeaux, Talence Cedex, Bordeaux, 33405 France

**Keywords:** High intensity focused ultrasound, Therapy guidance, Motion correction

## Abstract

**Background:**

During lengthy magnetic resonance-guided high intensity focused ultrasound (MRg-HIFU) thermal ablations in abdominal organs, the therapeutic work-flow is frequently hampered by various types of physiological motion occurring at different time-scales. If left un-addressed this can lead to an incomplete therapy and/or to tissue damage of organs-at-risk. While previous studies focus on correction schemes for displacements occurring at a particular time-scale within the work-flow of an MRg-HIFU therapy, in the current work we propose a motion correction strategy encompassing the entire work-flow.

**Methods:**

The proposed motion compensation framework consists of several linked components, each being adapted to motion occurring at a particular time-scale. While respiration was addressed through a fast correction scheme, long term organ drifts were compensated using a strategy operating on time-scales of several minutes. The framework relies on a periodic examination of the treated area via MR scans which are then registered to a reference scan acquired at the beginning of the therapy. The resulting displacements were used for both on-the-fly re-optimization of the interventional plan and to ensure the spatial fidelity between the different steps of the therapeutic work-flow. The approach was validated in three complementary studies: an experiment conducted on a phantom undergoing a known motion pattern, a study performed on the abdomen of 10 healthy volunteers and during 3 in-vivo MRg-HIFU ablations on porcine liver.

**Results:**

Results have shown that, during lengthy MRg-HIFU thermal therapies, the human liver and kidney can manifest displacements that exceed acceptable therapeutic margins. Also, it was demonstrated that the proposed framework is capable of providing motion estimates with sub-voxel precision and accuracy. Finally, the 3 successful animal studies demonstrate the compatibility of the proposed approach with the work-flow of an MRg-HIFU intervention under clinical conditions.

**Conclusions:**

In the current study we proposed an image-based motion compensation framework dedicated to MRg-HIFU thermal ablations in the abdomen, providing the possibility to re-optimize the therapy plan on-the-fly with the patient on the interventional table. Moreover, we have demonstrated that even under clinical conditions, the proposed approach is fully capable of continuously ensuring the spatial fidelity between the different phases of the therapeutic work-flow.

## Background

Percutaneous thermal ablation of tumors has emerged as an alternate treatment option for patient groups affected by unresectable pathologies and/or are not eligible for surgical interventions [1]. Such therapies rely on locally increasing the temperature of the pathological tissue to an extent that induces irreversible cell injury and eventually apoptosis and/or coagulative necrosis [2]. In particular, high intensity focused ultrasound (HIFU) [3–5] is currently the only percutaneous thermal ablation modality capable of non-invasive treatment delivery [1, 6]. It has already met success in treating several medical conditions such as: palliation of painful bone metastases, uterine fibroids, prostate malignancies, liver tumors and several neurological diseases such as tremor-dominant Parkinson’s or neuropathic pain [7]. However, HIFU thermal ablations still remain challenging when the target pathology is situated in the abdomen or lower thorax. The challenge mainly stems from the fact that therapy delivery in such areas is hampered by various types of physiological motion, occurring at different time scales [8–10]:

- Respiratory motion, for example, leads to a rapid quasi-periodic displacement of the organs in the upper abdomen and thoracic cage, with a typical frequency of 0.2 - 0.3 Hz (3 - 5 s per respiratory cycle)[11]. Previous studies have addressed this type of motion through different compensation techniques such as breath-holding, gating and/or beam-steering, with each approach involving their own set of advantages and drawbacks [12].
- Digestive activity, metabolic processes and muscle relaxation have been identified to lead to significant displacements of abdominal organs on time scales of several minutes [13–15]. For this reason, in the scope of this paper, such motion will be referred to as slow physiological/long term drifts. The term “drift” was chosen due to the fact that contrary to respiration, these types of motion are generally of progressive nature and irreversible. The problem of digestive and peristaltic activity, in particular, can be alleviated by adjusting the patient’s diet prior to the intervention [16] or by the administration of drugs such as butylscopolamine and/or glucagon acting as digestive motility inhibitors [17, 18]. Long term drifts originating from other physiological sources such as bladder filling are usually addressed by the use of Foley catheters [19, 20].
- Finally, spontaneous motion due to, for example, muscle spasms is fast and infrequent, making it difficult to predict and to compensate for. It becomes particularly problematic for long interventions in absence of sedation or anesthesia, requiring the patient to lie in an uncomfortable position for lengthy periods of time. This is usually addressed by using molds or casts or by putting the patient under sedation [21, 22].

If left un-addressed, motion can lead to the therapeutic energy being diverted from the anatomy due for ablation. This not only increases the risk of under-treating the pathology but can also lead to unnecessary damage to otherwise healthy tissue [9, 23, 24]. Thus, a motion compensation strategy dedicated to HIFU thermal ablations in the abdomen can be beneficial for reducing the probability of such developments.

Regardless of the approach used for thermal ablation, there are multiple imaging modalities that can be used for therapy guidance [12, 25–28], with magnetic resonance imaging (MRI) possibly being the most versatile. Besides allowing precise delineation and identification of the pathology due to its superior soft tissue contrast [29], it also provides a means to non-invasively monitor in real-time the temperature of the treated anatomy and its surroundings through a technique called MR-thermometry [30]. The MR temperature measurements are typically the main observable during MR-guided HIFU (MRg-HIFU) thermal ablations. By computing the time integral of a non-linear temperature dependent term at a particular anatomical location, thermal dose measurements can be obtained [31], providing a mean to quantify thermal damage. The unit of measurement for thermal dose is equivalent minutes at 43°C (CEM_43_), with an anatomy being regarded as necrotic once it exceeds 240 CEM_43_ [31, 32]. However, motion-induced spatial misalignments between the MR temperature maps will most likely lead to miscalculations of the delivered thermal dose, since the associated time integral at a point in space actually includes temperature measurements from different anatomical locations. Thus, a motion compensation strategy which ensures the spatial alignment between the MR temperature maps is expected to improve the monitoring of therapy progress and effectiveness. Moreover, MR-thermometry acquisition sequences that are optimized for acquisition speed, which is usually the case for MRg-HIFU therapies, are often subject to geometric distortions [33]. This leads to a spatial inconsistency between the apparent location of a voxel in the acquired image and its true position in the imaged anatomy. Thus, a spatial misalignment between the temperature maps and the true underlying anatomy may occur. Therefore, it would be preferable that the geometric distortions, which can potentially affect the MR-thermometry images, are addressed prior to the calculation of the thermal dose.

While previous studies concerned with motion compensation during MRg-HIFU thermal therapies focused on displacements occurring at a particular time-scale [12, 34], in the current study we propose a motion correction framework that encompasses the entire intervention. Our solution consists of several linked motion compensation modules, each addressing a particular type of displacement/deformation, including:

1. A correction scheme for slow physiological motion, allowing on-the-fly adaptation of the interventional plan according to the displacements exhibited by the target anatomy.
2. A respiratory motion compensation scheme operating during HIFU energy deliveries, which ensures the spatial alignment between temperature measurements. This, in turn, is expected to lead to a more accurate evaluation of the delivered thermal dose.
3. A feature which allows therapy progress to be evaluated on-the-fly, in a spatially consistent way, on the interventional planning image(s) acquired at the beginning of the therapy.
4. A method for correcting the geometric distortions that frequently affect the MR-thermometry images, ensuring a spatial consistency between the estimated thermal dose maps and the true underlying anatomy.

The above features were integrated in the work-flow of an MRg-HIFU thermal ablation, ensuring continuous availability of the target position over the duration of the intervention. For the remainder of the manuscript percutaneous thermal ablations by the means of HIFU will be simply referred to as HIFU therapies/interventions/ablations.

## Methods

### General strategy

**Fig. 1:**
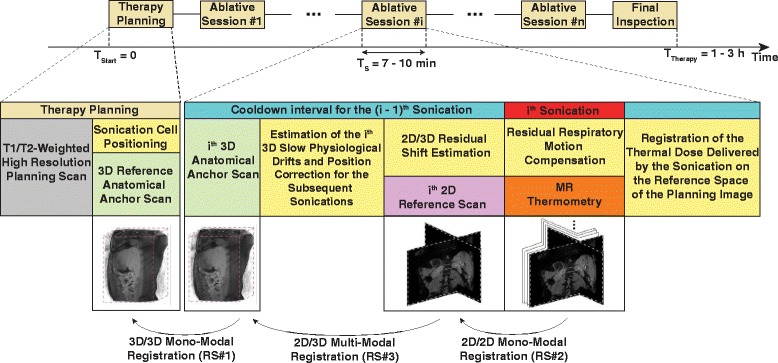
Proposed motion estimation framework for continuous target tracking during MRg-HIFU interventions

Figure 1 illustrates schematically the typical work-flow of an MRg-HIFU therapy, together with the proposed motion correction framework. The work-flow of an MRg-HIFU thermal therapy is in practice frequently episodic: bursts of energy deliveries (called sonications) are interleaved with periods of inactivity, during which the tissues in the near- and far-field are allowed to cool down. This allowed various MR-scans to be integrated at different stages of the therapy, which had the purpose of sampling the position of the treated anatomy and its surroundings over the duration of the intervention. The scans include 3D acquisitions several minutes apart (green boxes in Fig. 1), for sampling long-term drifts, while motion during sonications was sampled using more rapidly acquired 2D MR images, primarily used for thermometry (orange box in Fig. 1). Motion estimation was achieved by comparing the acquired MR-images, using image registration [35], to a reference image acquired at the beginning of the therapy. In order to relate all the acquired MR images to a single reference, several registration steps were employed: 1) A 3D-to-3D registration step (RS *#*1), for estimating long term drifts; 2) A 2D-to-2D registration step (RS *#*2), for estimating motion during sonications; 3) A 2D-to-3D registration step (RS *#*3), for estimating any residual displacement between a 2D reference image (pink box in Fig. 1) and its preceding 3D volume. All of these steps and the manner in which they link to each other will be described in detail during later sections.

The design of the framework includes images of two different MR contrasts: one for the 3D and one for the 2D images. For this reason, image registration was performed using two classes of algorithms: mono- and multi-modal [36]. In the scope of this study, a mono-modal registration method, namely the optical flow algorithm [37], was employed when the compared images had the same contrast weighting (more specifically during (RS *#*1) and (RS *#*2)). For images acquired with different contrasts, a multi-modal method based on the modality independent neighborhood descriptors (MIND) [38] was used (more specifically during (RS *#*3)). An important feature of both the optical flow and the MIND algorithm is their capability of providing dense and elastic deformations. This is particularly beneficial for estimating the complex deformations underwent by abdominal and thoracic organs.

The resulting estimated displacements/deformations provided by the framework were used for two purposes:

1. A “down-stream” propagation of the planned sonication positions such that their updated location match the initial anatomy due for ablation.
2. An “up-stream” propagation of the thermal dose delivered by each individual sonication such that therapy progress evaluation can be made in a common reference-frame.

Both the MR-acquisitions and the energy deliveries were performed using respiratory gating via a pencil-beam navigator placed on the diaphragm [39]. This implied that images were acquired and/or the HIFU beam was turned on only when the diaphragm is close to a predefined range of locations, referred to as the gating window.

#### Estimation of the 3D slow physiological drifts

In order to measure the long term drifts of the target area and its surroundings, a T1-weighted 3D scan is acquired after each sonication (green boxes in Fig. 1), during the cool-down intervals. The drifts are then estimated by registering the 3D images via the optical flow algorithm, to a reference scan of the same size and contrast acquired at the beginning of the therapy (RS *#*1).

The 3D scans employed the following MR acquisition protocol: TE = 2ms, TR = 4.3ms, image matrix 192×192×75, 10° flip angle, with an isotropic voxel size of 2×2×2 mm^3^, resulting in an acquisition time of 60 − 90 s, depending on the frequency and reproducibility of the subject’s breathing cycle. For the remainder of the manuscript, this type of images will be referred to as 3D anatomical anchors.

#### Real-time compensation of respiratory motion

Respiratory gating was used as a first-order method for respiratory motion compensation and is expected to considerably reduce the side-effects of respiration during individual sonications, since energy deposition becomes more localized. However, due to the finite size of the gating window, residual respiratory motion might still be present. This can lead to spatial misalignments between the MR temperature maps and in turn to miscalculations of the thermal dose.

In order to estimate the residual displacements between the temperature maps, the magnitude images provided by MR-thermometry during a particular sonication (orange box in Fig. 1) were registered in real-time (RS *#*2), using the optical flow algorithm, to a common reference scan (pink box in Fig. 1). The resulting motion estimates were then used to spatially align the temperature measurements, thus more accurate thermal dose estimates are expected.

MR-thermometry was performed using the proton resonance frequency shift (PRFS) [30], with the acquisition sequence employing the following parameters: each scan was a single shot gradient-recalled echo, TE = 15 ms, TR = 72.5 ms, 20° flip angle, image size 160×160, voxel size 2.5×2.5×7 mm^3^. Concerning spatial coverage, each dynamic consisted of one coronal and one sagittal slice intersecting in the focal point. All the scans in the MR-thermometry series were acquired with a 5 mm gating window, except the 2D reference scan used in the registration process (pink box in Fig. 1). For the latter, the gating window was reduced to 2 mm for increased precision, which will be explained later in the manuscript.

#### Registration of the thermal dose measurements to a common reference

Correcting the temperature maps with respect to respiratory motion should provide improved thermal dose measurements for a particular sonication. At this point, however, each MR-thermometry series is registered to their own dedicated reference image. This leads to the resulting individual thermal dose maps being represented in their own frame-of-reference. It is, however, preferable to have all thermal dose measurements mapped into the reference frame of the planning image. In this manner, therapy progress can be monitored relative to the interventional plan, in a spatially consistent way. This mapping of the thermal dose measurements was achieved in two steps. First, the 2D reference scan was registered to its preceding 3D anatomical anchor (RS *#*3), in order to account for any potential residual displacements. This was followed by a mapping of the thermal dose with respect to both these residual displacements and the 3D drifts estimated on that particular anatomical anchor, thus projecting the dose into the reference space of the planning image.

The 2D reference scan and the 3D anatomical anchor have different dimensionality, geometry and MR-contrast weighting. In effect, their registration was achieved via a two-step process. Initially, the geometry and dimensionality issue was addressed by re-formatting the 3D anatomical anchor into the coordinate system of the 2D reference scan. The re-formatting was performed by relying on geometrical information extracted from the imaging parameters (field-of-view position, orientation, size, etc.). Once the 3D anchor was re-formatted, the planes covered by the 2D reference scan were extracted from the 3D re-formatted image and the alignment was further refined via the MIND multi-modal registration algorithm [38].

#### Extensions to the proposed motion correction framework

**Fig. 2:**
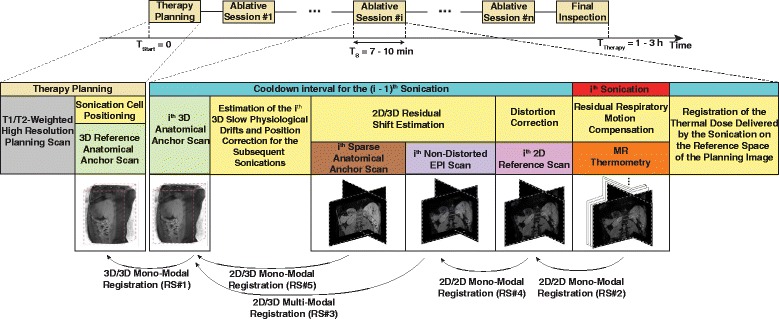
Extended motion estimation framework. In addition to the original, the extended version also includes a distortion correction scheme and a validation component for the multi-modal registration algorithm

Two additional components were included in the proposed framework, which do not directly contribute towards motion compensation, having instead validation purposes. The extended framework is displayed in Fig. 2. Note that it includes two additional scans: one called a non-distorted EPI (purple box) and the other a sparse anatomical anchor (brown box). The non-distorted EPI scan is used in the process of estimating the geometric distortions that frequently hamper fast MR thermometry acquisitions, while the purpose of the sparse anatomical anchor is in the validation of the MIND multi-modal registration algorithm. The two added components are described in more detail within the following two paragraphs.

##### Estimation of geometric distortions

In the proposed protocol, MR-thermometry is based on images acquired using an echo-planar imaging (EPI) readout train. In order to have fast temperature updates, the sequence was optimized for acquisition speed, which leads to the images containing geometric distortions [33, 40]. The latter manifest themselves as a mismatch between the apparent position of the anatomy in the acquires images and its true position. Thus, when registering the 2D reference scans to their preceding 3D anatomical anchors (RS *#*3 in Fig. 1), the estimated displacements will also include the geometric distortions.

In order to differentiate motion from geometric distortions we propose acquiring an additional image, which was called a non-distorted EPI scan (purple box in Fig. 2), having the same contrast and geometry as the 2D reference scans, but re-optimized such that distortions are minimized. An estimation of the geometric distortions is then obtained by registering via the optical flow algorithm the 2D reference scan to the non-distorted EPI (RS *#*4 in Fig. 2). The actual residual motion between the 2D reference scans and their preceding 3D anatomical anchor is now estimated using the non-distorted EPI (RS *#*3 in Fig. 2). The non-distorted EPI was acquired using a 2 mm gating window, immediately prior to the 2D reference scan. This also justifies why the latter was also acquired using a 2 mm gating window. Since the goal is to estimate solely geometric distortions, the potential residual motion between the 2D reference and the non-distorted EPI scans was minimized by using a narrow gating window.

##### Validation of the MIND multi-modal registration algorithm

Multi-modal algorithms tend to be more complex and error prone than mono-modal methods. Therefore, an independent validation procedure is proposed in the current work for the MIND algorithm. This procedure consists in the acquisition of an additional image immediately prior to the non-distorted EPI scan, in the same geometry, but with the contrast of the 3D anatomical anchors. The newly acquired image, called a sparse anatomical anchor (brown box in Fig. 2), is then registered to the 3D anatomical anchor via the optical flow algorithm (RS *#*5 in Fig. 2). This is performed, however, after re-formatting the 3D anatomical anchor into the coordinate system of the sparse anatomical anchor, and the selection of the corresponding planes. By design, the motion fields obtained during RS *#*3 and RS *#*5 should be identical. The deformations provided by the optical flow algorithm during RS *#*5 were established as a silver standard, with the errors associated to the MIND algorithm during RS *#*3 being quantified in terms of the endpoint error (EE):

1 $$ \text{EE}(\vec{r}) = \|\mathbf{u}_{\text{OF}}(\vec{r}) - \mathbf{u}_{\text{MIND}}(\vec{r}))\|_{2}   $$

where $\vec {r}$ is the pixel position, ∥·∥_2_ is the Euclidean distance and **u**_OF_ and **u**_MIND_ are the motion vectors estimated by the optical flow and MIND algorithms, respectively.

#### Registration algorithms

As previously mentioned, depending on whether the images being registered were acquired with the same MR-contrast weighting or not, one of two registration algorithms were employed: the optical flow [37] or the MIND [38] algorithm. The methods were chosen due to their fast numerical schemes, low number of input parameters and their capability to provide deformations on a voxel-by-voxel/pixel-by-pixel basis, characteristics which make them particularly attractive for medical image registration.

The optical flow algorithm followed the implementation described by Zachiu et al. [37]. The approach provides the deformation between two images *I* and *J*, as the minimizer of the following functional:

2 $$ E_{\text{OF}}(\mathbf{u}) = \sum_{\vec{r}\in\Omega}{\left(|I(\vec{r}) - J(\vec{r} + \mathbf{u}(\vec{\mathbf{r}}))| + \alpha\|\vec{\nabla}\mathbf{u}(\vec{\mathbf{r}})\|_{2}^{2}\right)}  $$

where **u** is the 2D or 3D displacement, depending on the image dimensionality, *Ω* is the image domain, $\vec {r}$ is a pixel/voxel spatial location, $\vec {\nabla }$ is the gradient operator, ∥·∥_2_ is the Euclidean norm and *α* is a parameter linking the two terms of the functional. The optimization scheme and method validation are discussed at large in the original paper [37].

The MIND algorithm, initially proposed by Heinrich et al. [38], is a deformable multi-modal/cross-contrast registration algorithm relying on the concept of self-similarity introduced by Buades et al. [41]. The method associates to each pixel/voxel of an image *I*, a descriptor based on local similarities defined by:

3 $$ \text{MIND}(I,\vec{r},\gamma) = \frac{1}{Z}\text{exp}\left(-\frac{D_{p}(I, \vec{r}, \vec{r} + \gamma)}{V(I, \vec{r})}\right)   $$

where *Z* is a normalization constant, *D*_*p*_ is the Euclidean distance between the local neighborhoods of size *p* around the pixels/voxels at positions $\vec {r}$ and $\vec {r}+\gamma $, *γ*∈*Γ* is a search region of the pixels/voxels included in the descriptor and $V(I, \vec {r})$ is a local variance estimation accounting for noise perturbations. In effect, a MIND descriptor associates to each pixel/voxel of the image *I* a vector of size *Γ*. According to the MIND algorithm, the deformations between two images are found as the minimizer of the following functional:

4 $$  E_{MIND}(\mathbf{u}) = \sum_{\vec{r}\in\Omega}{\left(S(I(\vec{r}), J(\vec{r}+\mathbf{u}(\vec{\mathbf{r}}))))^{2} + \alpha\|\vec{\nabla}\mathbf{u}(\vec{\mathbf{r}})\|_{2}^{2}\right)}  $$

with

5 $$ S(I, J) = \frac{1}{|\Gamma|}\sum_{\gamma\in\Gamma}{|\text{MIND}(I, \vec{r}, \gamma) - \text{MIND}(J, \vec{r}, \gamma)|}  $$

where *I* and *J* are the images to be registered. The optimization scheme together with implementation and validation details can be found in the original paper [38].

### Validation of the proposed motion estimation framework

The proposed motion estimation framework was validated in three complementary studies:

1. An experiment carried-out on a phantom undergoing a known motion pattern.
2. A study conducted on the abdomen of 10 healthy volunteers.
3. An in-vivo study involving MRg-HIFU thermal ablations on porcine liver, which included a total of 3 animal experiments.

#### Phantom experiment

**Fig. 3:**
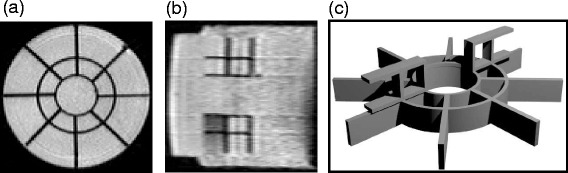
The custom-built phantom. **a**: A coronal and **b**: a sagittal slice of a T1-weighted 3D MR image acquired on the phantom. The two structures integrated in the phantom have a dark appearance in the images, while the polymer gel appears as bright. **c**: A 3D computer-generated rendering of the two structures inside the phantom

The motion estimates provided by the proposed framework were initially validated on a dataset acquired during a phantom study. Motion estimation algorithms typically rely on structural information in order to estimate displacements, having to interpolate/extrapolate/infer motion in more homogenous regions. For this reason, the phantom consisted of a set of two grid-like structures (positioned in the coronal and the sagittal plane) placed inside a cylindrical plastic casing. The casing itself was then filled with a polymer gel and one of the bases was provided with an acoustic-transparent mylar membrane. Figure 3(a) and (b) illustrate a coronal and a sagittal slice of a T1-weighted 3D MR image acquired on the phantom, showcasing the two integrated structures surrounded by the polymer gel. The 3D rendering in Fig. 3(c) displays the shape and the manner in which the two structures are positioned with respect to one-another.

Known displacements were induced to the phantom and used as gold-standard during the validation process. The known displacements were applied to the phantom via a motorized platform linked to an in-house developed interface which allowed the injection of custom-designed motion patterns. For the purpose of making the experiment more realistic, a pre-recorded free-breathing pattern was induced to the phantom. The pattern was recorded during a separate experiment and consisted of the average head-foot liver displacement of a healthy volunteer.

The phantom, moving according to the pre-recorded breathing pattern, underwent the extended MR image acquisition protocol illustrated in Fig. 2. A total of 9 3D anatomical anchors (including the reference) were acquired on the phantom, with all the other scans in between, over a duration of ∼1 h. During the experiment, two 5 mm drifts were induced in the breathing pattern after the acquisition of the 3^rd^ and respectively 6^th^ anatomical anchor, in order to simulate the effect of slow physiological motion. Note that the FOV of the 3D anatomical anchors was fixed in such a way that the phantom did not leave it following the two induced drifts.

Besides validating the motion estimates provided by the optical flow and the MIND algorithms, the phantom experiment also allowed evaluating the extent of the geometric distortions present in the 2D thermometry images and the performance of the proposed distortion correction scheme. For this purpose, the position of the grid points contained by the two structures placed inside the phantom, was manually identified in the non-distorted EPI scans and compared, in terms of the Euclidean distance, to their position in the succeeding 2D reference scans, before and after correction. The distances before and after correction, were then placed in an individual set, and the statistical distribution of the resulting two sets were compared in order to determine the extent to which the geometric distortions were corrected by the proposed scheme.

#### Healthy volunteer study

A study satisfying the required standards and in conformity with regulatory requirements was carried-out on 10 healthy volunteers. The main purpose of this experiment was to evaluate the displacements underwent by the human liver and kidneys over the typical duration of an MRg-HIFU intervention. Five of the volunteers were subjected to the original MR-protocol illustrated in Fig. 1, while the other five were put through the extended protocol displayed in Fig. 2. The volunteers were placed in the MR-scanner in a prone-head-first position and were instructed not to move over the duration of the study. Each experiment lasted for a maximum of 60 min, with a minimum duration imposed by the volunteer. During this time interval, the original or the extended (depending on the volunteer) MR-acquisition protocol was run continuously, with a time gap of 7-8 min between the 3D anatomical anchors.

The 3D slow physiological drifts and the residual respiratory motion present within the MR-thermometry series were quantified for the liver and kidneys of each individual volunteer. The quantification was performed in terms of the spatial and/or statistical distribution of the following set:

6 $$  M = \{\|\mathbf{u}(\vec{r})\|_{2}^{2}\, | \, \vec{r}\in\text{ROI}\}  $$

where **u** are the estimated displacements, $\vec {r}$ is the spatial position, ∥·∥_2_ is the Euclidean norm and ROI is a region encompassing an organ of interest. Basically, *M* is a set containing the magnitude of the motion vectors estimated in all pixels/voxels belonging to the organs of interest. Such a metric is meant to provide the extent of the deformations undergone by the organs. Additionally, by using the metric in Eq. 1, validation of the motion estimates provided by the MIND cross-contrast registration algorithm was performed for the volunteers that underwent the extended MR-protocol.

In order to define the ROI in Eq. 6 encompassing the organs of interest, an active contour-based segmentation procedure was employed, which was subsequently manually refined. The segmentation was performed using ITK-Snap v3.0 [42].

#### In-vivo experiments

The current animal study was performed in agreement with the European law on animal experimentation and in compliance with the institution’s rules for animal care and use.

In order to evaluate the compatibility of the proposed motion estimation framework with the work-flow of an MRg-HIFU intervention under clinical conditions, 3 animal experiments were conducted, consisting of in-vivo ablations on porcine liver. Similar to the study performed, for example, by Wijlemans et al, the MRg-HIFU ablations were carried-out on female Dalland pigs of 60 – 70 kg. The animals were anesthetized by an initial intramuscular injection containing ketamine (13 mg/kg), midazolam (0.7 mg/kg), atropine (0.02 mg/kg) and meloxicam (0.4 mg/kg) and placed under mechanical ventilation. Subsequently, general anesthesia was maintained by continuous intravenous administration of sufentanil (11.3 *μ*g/kg/h), midazolam (1 mg/kg/h) and cisatracurium (0.09 mg/kg/h). A total number of 6 sonications were performed on one of the animals and 9 on the other two. The sonication cells had a 4 mm diameter, and each sonication consisted in delivering 450 W of acoustic power over a duration of ∼30 s, resulting in ∼13.5 kJ of energy per shot. The sonications were carried-out at a frequency of 1.2 MHz and a depth of ∼10 cm, using a modified Philips Sonalleve ablation system (Philips Healthcare, Vantaa, Finland). Time-wise, the experiments extended over a duration of ∼1-2h each.

In order to prevent rather large delays during the experiments that would allow naturally occurring slow physiological drifts to become significant, artificial motion was induced in the abdominal area of the animals. This was achieved by varying the volume of water within a cooling cushion placed between the mylar membrane of the Philips Sonalleve system and the skin of the animals. Technical details related to the cushion can be found in [43] and Chapter 6 of [44]. Throughout each experiment, the volume of water inside the cooling cushion was varied twice, reducing the cushion’s height by ∼5 mm each time.

During animal experiment *#* 3, immediately after the last sonication, an additional 3D anatomical anchor was acquired, followed by a contrast-enhanced (CE) 3D T1w scan. The additional 3D anchor was registered to the reference 3D anchor and the resulting deformations were used to map the CE 3D T1w image into the reference space of the planning image. In this manner, the non-perfused volume (NPV) visible on the CE image, can be compared to the initial volume due for ablation and the up-stream propagated thermal dose, in a spatially consistent way. The CE 3D T1w acquisition sequence employed the following parameters: TE = 2.6ms, TR = 5.4ms, image matrix 512×512×53, 10° flip angle, with a voxel size of 0.48×0.48×1.5 mm^3^. Prior to its mapping into the reference space of the planning image, the CE 3D T1w scan was reformatted onto the grid of the 3D anatomical anchors. This facilitated the consistent application of the deformation estimated on the last 3D anatomical anchor. Once the experiments were finished, the animals were euthanized using an overdose of sodium pentobarbital.

### Hardware and implementation

A multi-threaded (8 threads) C++ implementation was performed for all registration algorithms included by the proposed motion correction framework. The data resulting from the healthy volunteer study and the phantom experiment was processed in retrospect, with motion estimation and analysis being conducted on an Intel 3.2 GHz i7 workstation (8 cores) with 16 GB of RAM.

During the animal experiments, the calculations associated to the proposed framework were offloaded on a dedicated custom-build node with 32 cores and 64 GB of RAM. The implementation was performed as an additional module directly into the clinical software dedicated to the interventional radiologist delivering the therapy. The node together with access to the clinical software code was provided by Philips Healthcare, Vantaa, Finland.

## Results

### Phantom study

#### Validation of the optical flow mono-modal registration algorithm

**Fig. 4:**
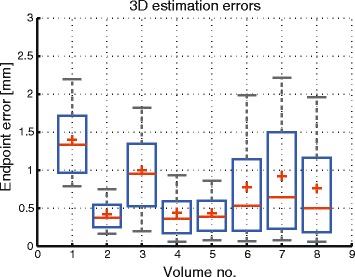
Validation of the optical flow algorithm on the phantom dataset. The boxplots correspond to the statistical distribution of the EE between the estimated motion vectors and the injected drifts for each 3D anatomical anchor acquired during the phantom experiment. The abscissa provides the index of the 3D anatomical anchor, for which the errors are displayed, within the corresponding time-series

A total of 9 volumes (including the reference 3D anchor) were acquired on the phantom undergoing a known motion pattern, with a 5 mm drift injected in the pattern after every third scan. In order to validate the optical flow mono-modal registration algorithm, the magnitude of the motion vectors estimated on the 3D anchors were compared to the injected drifts. The resulting EE between the estimated and the injected displacements are displayed in Fig. 4. The illustrated boxplots correspond to the statistical distribution of the errors estimated on each of the 3D images (except the reference scan itself). The boxplots were constructed as follows: the box limits are the 25^th^ and the 75^th^ percentiles, the whiskers correspond to the 5^th^ and the 95^th^ percentiles, the red cross indicates the average of the set and the red line is the set median. Note that, for most of the volumes, 95% of the errors remain sub-voxel (<2 mm). It is only for two of the volumes that the 95^th^ percentile of the registration errors marginally exceeds this threshold. Also, with one exception (the first of the volumes), the average EE remains sub-millimeter. The analysis was restricted to the structures included in the body of the phantom.

#### Validation of the multi-modal registration algorithm

**Fig. 5:**
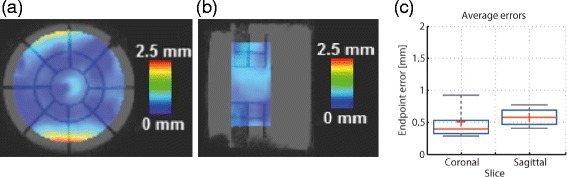
Validation of the multi-modal registration algorithm on the moving phantom data set. The spatial distribution of the temporally averaged EE in a (**a**): coronal (**b**): sagittal plane through the phantom (**c**): Statistical distribution of the errors illustrated in (**a**) and (**b**)

A validation of the motion estimates provided by the MIND multi-modal registration algorithm was also performed on the phantom dataset. However, instead of registering the non-distorted EPI image to its preceding 3D anatomical anchor, it was registered directly to the 3D reference anchor (see Fig. 2 for reference). The estimated motion vectors were then compared in terms of the EE to the drifts induced in the motion pattern. The spatial distribution of the temporally averaged EE maps is displayed in Fig. 5(a) and (b) for a coronal and a sagittal slice respectively. It can be observed that the EE remain below the in-plane voxel size (<2.5 mm). This is further confirmed by analyzing their statistical distribution illustrated in Fig. 5(c). Moreover, the two boxplots in Fig. 5(c) show that 95% of the errors remain sub-millimeter with an average of ∼0.5 mm. Note that the analysis was restricted to the two structures embedded in the phantom. The borders of the phantom were also excluded from the analysis, due to a signal drop in the non-distorted EPI images, particularly visible in Fig. 5(b).

#### Quantification and correction of the geometric distortions present in the MR-thermometry images

**Fig. 6:**
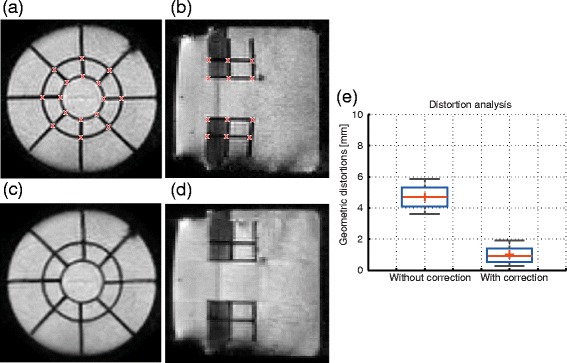
Validation of the proposed distortion correction scheme employed for the MR-thermometry images. Example of a coronal (left) and a sagittal (middle) MR-thermometry magnitude image acquired on the phantom (**a**), (**b**): before and (**c**), (**d**): after distortion correction. (**e**): Extent of the geometric distortions measured for the grid points marked with “x” in **a** and **b**, before (left boxplot) and after (right boxplot) correction

Figure 6(a) and (b) display a coronal and a sagittal slice from an MR-thermometry image acquired on the phantom, before distortion correction. It can be observed that some segments of the structures inside the phantom appear to be bent, when in reality the structures are made of straight elements. This effect is notably reduced after distortion correction, as shown in Fig. 6(c) and (d).

The grid-like shape of the structures integrated in the body of the phantom, in both the coronal and sagittal plane, allowed the quantification of the geometric distortions present in the MR-thermometry images and also the extent to which these are corrected by the proposed method. The boxplots in Fig. 6(e) display the extent of the geometric distortions before and after applying the proposed correction scheme. Measurements were performed for the grid points marked with “x” in Fig. 6(a) and (b), for all the 2D reference - non-distorted EPI pairs acquired on the phantom. It can be observed that, in the absence of correction, distortions extend up to ∼6 mm. After correction, however, distortions were reduced to in-plane voxel size values (<2.5 mm), with an average reduced from ∼5 mm to ∼1 mm.

### Volunteer study

#### Analysis of the 3D slow physiological drifts

**Fig. 7:**
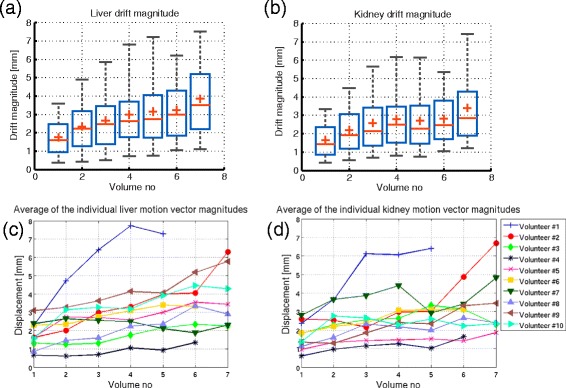
Temporal evolution of the 3D slow physiological drifts estimated on the 10 healthy volunteers. **a**, **b**: Statistical distribution of the magnitude of the 3D motion vectors estimated on the liver and kidneys, pooled from all volunteers, at each 3D anatomical anchor acquisition time point. **c**, **d**: Time evolution of the average magnitude of the motion vectors estimated on the liver and kidneys, illustrated individually for each of the 10 healthy volunteers. The abscissa in Fig. **a** - **d** provides the index of the 3D anatomical anchor, for which the displacements are displayed, within the time-series

Figure 7 illustrates the temporal evolution of the long term drifts estimated on the 10 healthy volunteers. Figure 7(a) and (b) show, separately for the liver and kidneys, the statistical distribution of the magnitude of the 3D motion vectors, pooled from all volunteers, at each 3D anatomical anchor acquisition time point. It can be observed, for both the liver and kidneys, that there is a tendency of the displacements to increase over time, with a magnitude of the motion vectors exceeding 7 mm at the acquisition time point of the last 3D anatomical anchor. Figure 7(c) and (d) display separately for the liver and kidneys, the average magnitude of the displacement vectors over time, individually for each of the 10 volunteers. The different length of some curves compared to others is due to the respective volunteers terminating the experiment before the 60 min maximum observation period. A rather large inter-subject variability can be observed in both the liver and kidneys. In volunteer *#*4, for example, the average displacement remained under 2 mm for the entire duration of the study, for all organs of interest. However, in volunteer *#*1, already halfway through the experiment, the average displacement exceeded 6 mm.

#### Assessment of the residual respiratory motion present in the MR-thermometry series

**Fig. 8:**
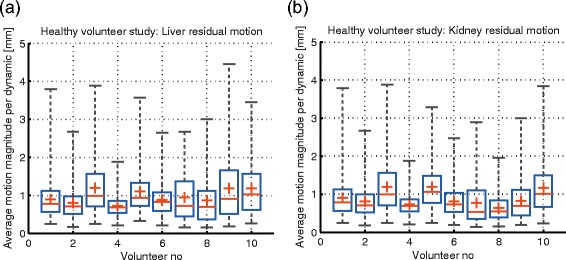
Quantification of the residual respiratory motion for the 10 healthy volunteers. Statistical distribution of the average magnitude of the motion vectors estimated for **a**: liver **b**: kidneys

For each dynamic of the multiple MR-thermometry series acquired on the healthy volunteers, the average liver and kidney residual respiratory displacement was estimated and pooled in a separate set for each volunteer. Figure 8(a) and (b) display, per individual, the statistical distribution of the average magnitude of the 2D motion vectors corresponding to the residual respiratory displacements, separately for the liver and the kidneys. While the average displacements remain close to 1 mm, they occupy a rather large range of values, in some instances exceeding 4 mm. The extent of the residual motion and the inter-individual variations are most likely determined by the stability/reproducibility of the breathing cycle of each individual volunteer.

#### Validation of the multi-modal registration algorithm

**Fig. 9:**
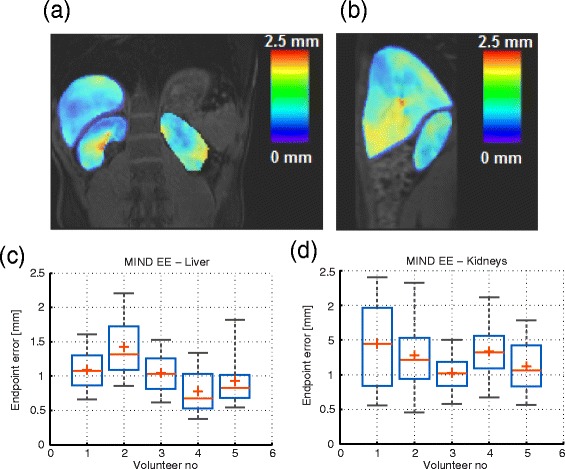
Validation, on the healthy volunteer dataset, of the MIND registration algorithm. **a**, **b**: Spatial distribution of the temporally averaged registration errors in the liver and kidneys of one of the volunteers, in a coronal and a sagittal plane respectively. **c**, **d**: Statistical distribution of the multi-modal registration errors in the liver and kidneys of each volunteer

As specified in the methods section, in order to validate the MIND registration algorithm, an endpoint error map (see Eq. 1) was calculated for each sparse anatomical anchor - non-distorted EPI image pair acquired on the volunteers (see Fig. 2 for scan nomenclature). Figure 9(a) and (b) illustrate for one of the volunteers, the spatial distribution in the organs of interest of the temporally averaged EE maps. It can be observed that, for this particular volunteer, the estimation errors for the MIND multi-modal algorithm remain under 2.5 mm, which corresponds to the in-plane voxel size. The pixel-wise EE were pooled separately for the liver and kidneys of each volunteer and illustrated under the shape of a boxplot in Fig. 9(c) and (d). For all volunteers, the estimation errors associated to the MIND algorithm reside beneath the in-plane voxel size.

### Animal experiments

**Fig. 10:**
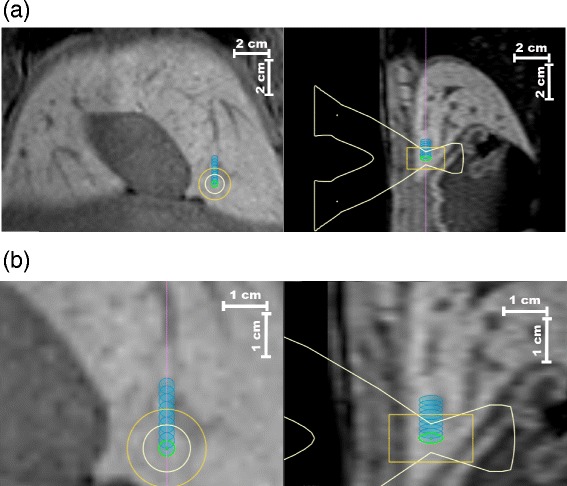
Therapy plan for animal experiment *#*3. Coronal (left) and sagittal (right) slice through the 3D planning image together with the 9 sonication cells **a**: overall **b**: magnified for better visibility

**Fig. 11:**
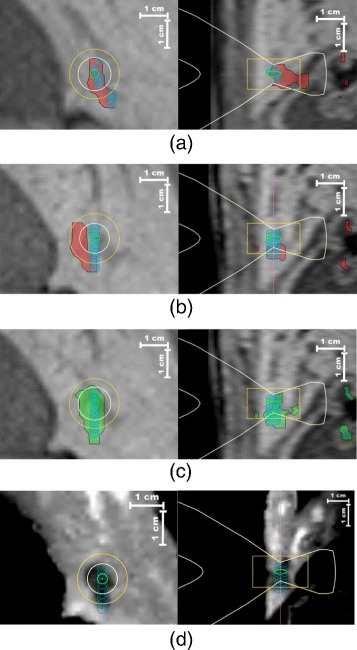
Validation of the proposed motion compensation framework under clinical conditions. Outcome of the MRg-HIFU liver ablation in animal experiment *#*3. **a**: Down-propagated/Motion corrected sonication locations (blue ellipses) overlapped with the non-registered lethal thermal dose (in red). **b**: Originally planned sonication locations (blue ellipses) overlapped with the non-registered lethal thermal dose (in red). **c**: Originally planned location of the sonications (blue ellipses) overlapped with the motion corrected lethal thermal dose (in green). **d**: The initially planned sonication cells (blue ellipses) overlaid on a coronal (left) and a sagittal (right) slice of the registered contrast-enhanced T1w image. Figures **a**, **b** and **c** all showcase a coronal (left image) and a sagittal (right image) slice through the 3D planning image acquired at the beginning of the therapy as background

The proposed motion correction framework was validated under clinical conditions during 3 separate in-vivo MRg-HIFU thermal ablations conducted on porcine liver. Figure 10(a) illustrates the therapy planning for animal experiment *#*3, showcasing a coronal (left) and respectively a sagittal (right) slice through the 3D planning image, upon which the 9 sonication cells are overlaid. For better visibility, the interventional plan was magnified and shown in Fig. 10(b). For this particular experiment, an artificial motion event was induced in the abdominal area of the animal after every 3 sonications. In effect, the initially planned sonication positions were updated twice during the experiment, according to the displacements estimated on the 3D anatomical anchors after each artificial motion event. Figure 11(a) illustrates the “down-propagated” sonication cells overlaid as blue ellipses on a coronal and a sagittal slice from the planning image. Following the two motion events, displacements of over 5 mm can be observed for all initially planned sonication cells. The red overlay represents the lethal thermal dose accumulated from all sonications, without mapping the thermal dose delivered by the individual sonications into the reference space of the planning image. This provides the means to evaluate the effects of motion on the outcome of the therapy, in the absence of the proposed motion compensation framework. This scenario is better illustrated in Figure 11(b) where the initial plan is overlaid as blue ellipses on the planning image, together with the non-registered lethal thermal dose in red. In the absence of a motion compensation strategy, a large part of the anatomy initially due for ablation would have been left untreated, while at the same time the therapy would have resulted in considerable collateral damage. Following the “up-stream” propagation and accumulation of the thermal dose delivered by each individual sonication, the result shown in Fig. 11(c) was obtained. The initially planned location of the sonications is displayed as blue ellipses overlaid on the interventional planning image, together with the motion corrected lethal thermal dose. The good overlap between the latter and the initial plan indicates that the proposed motion compensation framework performed as intended. Figure 11(d) displays the initially planned sonication cells overlaid as blue ellipses on a CE T1w image (acquired exclusively on animal *#*3), after its registration to the planning image. A good correspondence can be observed between the NPV, the initial volume due for ablation and the registered lethal thermal dose map displayed in Fig. 11(c). Note that the contrast of the Fig. 11(d) was digitally enhanced for improved NPV visibility. In all images from Figs. 10 and 11, the white overlay traces an approximation of the HIFU beam cone, while the yellow overlay defines a search region within which the HIFU system checks for the existence of a focal spot.

Table 1 reports, for the animal experiments, the percentage of the anatomical volume due for ablation that was estimated to receive a lethal amount of thermal dose, with and without enabling the proposed motion compensation framework. Although to different extents, when motion compensation is enabled, improvements in coverage were observed in all reported cases.

**Table 1:** Validation of the proposed motion compensation framework under clinical conditions. Percentage of the volume due for ablation estimated to have received a lethal amount of thermal dose with (first row) and without (second row) the proposed motion compensation framework enabled

	Animal *#*1	Animal *#*2	Animal *#*3
With motion correction	88%	74%	70%
Without motion correction	36%	63%	40%

### Computational performance of the registration algorithms

In order to ensure a smooth work-flow for the MRg-HIFU intervention, the registration algorithms included by the proposed framework should provide positional information with minimal latency. Table 2 reports the average convergence time for each of the registration steps included by the framework. See Fig. 1 for details concerning the purpose of each algorithm.

**Table 2:** Average convergence time per pair of images of the registration algorithms included by the proposed motion compensation framework

Step	Average convergence time [ms]
Estimation of 3D drifts (RS *#*1 in Fig. 1)	15000
Estimation of residual respiratory motion (RS *#*2 in Fig. 1)	80
Registration between the 2D reference image and its preceding 3D anchor (RS *#*3 in Fig. 1)	400

## Discussion

Lengthy MRg-HIFU thermal therapies in the abdomen are usually hampered by various types of physiological motion occurring at different time-scales. So far, studies have focused on developing correction schemes for displacements arising at a particular time-scale [12, 34] which, as we have demonstrated in the current work, may be conceptually insufficient. In effect, the present study proposes a motion compensation framework that encompasses the entire work-flow of an MRg-HIFU intervention. The framework consists of several linked components, each dedicated to estimating a particular type of motion/deformation, with the resulting displacements being used for two purposes: 1) Down-stream propagation of the initially planned sonication locations such that they match the current position of the anatomy and 2) Up-stream propagation of the thermal dose delivered by each individual sonication such that therapy progress can be evaluated in a single frame of reference (namely the reference space of the planning image(s)). Due to its modular nature, the proposed approach has increased flexibility, facilitating the addition, modification, replacement and/or removal of individual components. The proposed motion compensation strategy was tested and validated in three complementary experiments: 1) An experiment carried-out on a phantom undergoing a known motion pattern; 2) A study conducted on the abdomen of 10 healthy volunteers and 3) An in-vivo study involving HIFU ablations on porcine liver.

The healthy volunteer study reconfirmed that over a duration of 1h, the human liver and kidneys can manifest slow physiological drifts of up to 7 – 8 mm, exceeding acceptable therapeutic margins. This is in good correspondence with previous reportings [13, 45, 46]. Additionally, a rather large inter-subject variability was observed. Concerning the proposed correction scheme for respiratory motion, the study conducted on the 10 volunteers demonstrated that gating during energy deliveries, as a first order method, has good motion compensation capabilities, with estimated average residual displacement of ∼1-1.5 mm. Of importance are, however, the instances in which the estimated average residual displacements extend up to 4 mm or more (see Fig. 8). Due to the misalignments induced between the temperature maps, such displacements during MR-thermometry can impact the thermal dose measurements to an extent that they become unreliable for that particular sonication. A simple solution to reduce the range of residual respiratory displacements is to narrow-down the size of the gating window. However, depending on the reproducibility of the patient’s breathing cycle, this can lead to a poor duty cycle of the HIFU beam, affecting the overall therapy efficiency. The proposed dedicated registration scheme, on the other hand, allows energy deliveries with a wide gating window (≥5*m* *m*), facilitating a higher duty cycle not only for the HIFU beam, but also for the MR-thermometry.

The reliability of the motion estimates provided by the proposed framework was analyzed in both the phantom and the volunteer experiment. Following the phantom study, both the mono- and the multi-modal registration algorithms have proven on average sub-voxel accuracy and precision. In order to avoid a bias due to outliers during the validation of the multi-modal registration algorithm, the borders of the phantom were excluded from the analysis (see Fig. 5). This was performed due to a signal drop in these areas in the non-distorted EPI images, signal drops which were not present in the reference 3D anatomical anchor. Due to a violation of the basic assumption made by the MIND algorithm, that all structures in the reference image have a counterpart in the moving image, the reliability of the motion estimates was poor on the phantom borders. Since this aspect is known a priori, these low-signal areas are not representative for the algorithm’s performance. Instead they simply emphasize some of its limitations. The phantom experiment, however, has only limited validation capabilities since the phantom was able to undergo motion with fewer degrees of freedom than an actual abdominal organ. On the other hand, while the known motion patterns induced to the phantom can be used as a robust gold standard when analyzing the performance of the registration algorithms, obtaining a gold standard for in-vivo studies is a challenging task. In particular for the cross-contrast registration algorithm, this issue was addressed by comparing the in-vivo motion estimates against a silver standard. The latter was constructed based on motion estimates provided by the optical flow algorithm. This decision was made due to its prior successful in-vivo validation in previous independent studies [47–50]. However, the drawback of such an approach is that the optical flow algorithm has its own shortcomings which affect its performance (discussed at length in [37]). Thus, the in-vivo errors reported for the multi-modal algorithm stem from both its own mis-registrations and the errors in the silver standard itself. Nevertheless, the overall estimation errors remain sub-voxel for 3D registration and lower than the in-plane voxel size for the 2D registration methods, which is in good correspondence with previous studies [38, 47–50]. Potential errors may also occur during the registration of the 2D reference scan to its preceding 3D anatomical anchor (RS *#*3 in Fig. 1) or during the registration of the non-disorted EPI scan and the sparse anatomical anchor, again, to their preceding 3D anatomical anchor (RS *#*3 and RS *#*5 in the extended framework from Fig. 2). This is due to the fact that the elastic refinement of the registration between the 2D scans and the corresponding planes from the reformatted 3D anatomical anchor (following the initial rigid alignment step), was only performed in 2D. In case of severe through-plane motion, misregistration may occur, since through-plane motion might be interpreted as in-plane motion. The risk of such a development is, however, considerably reduced since the 3D anatomical anchor and the 2D reference scan, sparse anatomical anchor and the non-distorted EPI scan are respiratory gated and acquired in rapid succession. In the scope of this study, this led to residual displacements predominantly in the cranio-caudal direction, with the anterior-posterior component being well under the voxel size. For the estimation of through-plane deformations, a 3D dense and elastic registration between the 2D scans and their preceding 3D anatomical anchor would be necessary. This is, from a mathematical point-of-view, a severely ill-posed problem and a topic in itself, making it the object of future studies.

Since the proposed motion compensation framework consists of several linked components, with the estimated displacements in some instances being successively added to one another, error accumulation becomes an important aspect. While slow physiological drifts and residual respiratory displacements are the result of independent registrations between a scan and its corresponding reference image, with sub-voxel/sub-pixel estimation errors, projecting the thermal dose delivered by a particular sonication onto the reference space of the planning image relies on a chain of up to 4 registration algorithms (see Fig. 2). However, even so, the accumulated estimation errors remained within 10−20*%* of the total average displacement.

An effective way to improve the precision and accuracy of the registration algorithms is to increase the spatial resolution of the acquired MR images. A higher spatial resolution typically implies a greater level of detail and structural information in the images, which facilitates a better performance of the registration algorithms. Note, however, that MR imaging usually implies a tradeoff between spatial resolution, temporal resolution and signal-to-noise ratio (SNR). For a smooth work-flow of an MRg-HIFU therapy, constrains may have to be imposed on the image acquisition times. Therefore, a higher spatial resolution may result in a lower SNR of the acquired images. Particular attention is thus required, in order to ensure that the losses in terms of SNR do not counteract the gains in terms of precision and accuracy facilitated by a higher spatial resolution.

The overall performance of the proposed motion compensation framework together with its compatibility with the typical work-flow of an MRg-HIFU intervention under clinical conditions was validated during 3 animal experiments. In all 3 cases, the mapping of the thermal dose in the reference space of the planning image, with respect to the displacements estimated by the framework, resulted in different amounts of improvement between the planned sonication locations and the lethal thermal dose. Moreover, as shown in Fig. 11(c) and (d), for the animal for which a CE 3D T1w image was acquired at the end of the HIFU ablation session, a good correspondence can be observed between the planned location of the sonication cells, the registered lethal thermal dose and the registered NPV. This further confirms the success the proposed motion correction framework. Although in the scope of this study a CE T1w image was acquired only for the third animal, the visible NPV upon such images can generally be used as a metric for evaluating acute therapeutic response. Furthermore, in the context of motion correction schemes for MRg-HIFU, the NPV pattern can be used for additional validation, as demonstrated in animal experiment *#*3.

The majority of the MR-scans and registration algorithms included by the framework were integrated during the cool-down intervals between successive sonications. In order to ensure a smooth therapeutic work-flow, the acquisition times of the scans together with the computational requirements of the registration algorithms must not exceed typical cool-down durations. For the animal experiments, each sonication consisted in the delivery of ∼13.5 kJ of energy, which led to the HIFU system imposing cool-down intervals of 2-5 min, determined by the perfusion effects in the near-field. In practice, more typical values are 5 kJ of energy per sonication with 2-3 min cool-down. For the proposed framework, the duration of the MR-scans together with the registration algorithms integrated during the cool-down intervals resulted in average delays of 2-3 min, depending on the subject’s respiratory frequency. For the purpose of this study this was sufficient since such a duration is well in accordance with the cool-down threshold imposed by the HIFU system. Nevertheless, the protocol can be further accelerated if necessary. For example, this can be achieved by re-optimizing the acquisition sequence of the 3D anatomical anchors for speed rather than resolution and spatial coverage, which were favored in the current study.

In interventional oncology, pathology identification and delineation, interventional planning, therapy monitoring and therapy response evaluation are preferably performed in the same frame of reference. Especially in moving organs, this can become problematic for HIFU thermal ablations, since physiological motion frequently induces spatial mismatches between these steps. The current study aims to render the work-flow of an MRg-HIFU therapy in mobile organs compatible with pre-existing work-flows from interventional oncology by proposing a suitable motion estimation/correction strategy that encompasses all the previously specified phases of an intervention. Results have shown that physiological drifts of 7-8 mm have to be expected when therapy is conducted in the liver or kidneys, displacements which, if left unaddressed, can have severe consequences. For example, as illustrated in Fig. 11(b), in case the ablation area is situated in the proximity of the gallbladder, there is a high risk that the latter and/or the associated structures (such as the bile duct) are perforated/damaged, leading to complications due to the possible release of emulsifying enzymes into the bloodstream. Or, if therapy is conducted in the kidney, damage to the pelvis or the ureter might occur. Moreover, such displacements might lead to large areas of residual pathological tissue. Such a case is again depicted in Fig. 11(b), where only 40% of the initially planned anatomy would have been ablated (see also Table 1). However, the animal experiments have demonstrated that the risk of such developments can be considerably reduced when therapy is conducted with the proposed motion compensation framework active. As shown in Table 1, in all analyzed cases the framework led to improvements of the overlap between the planned anatomy due for ablation and the delivered lethal thermal dose, compared to the scenario when no corrections are performed. Moreover, the framework demonstrated good compatibility with the typical work-flow of an MRg-HIFU thermal therapy, with latencies introduced by the integrated MR scans and the registration algorithms that allowed a smooth progress of the intervention. Additionally, the fact that the proposed motion compensation strategy was implemented directly into the clinical software stack granted the possibility of on-the-fly re-optimization of the therapeutic plan according to the estimated displacements and the projection of the thermal dose delivered by each individual sonication in the same frame of reference (see Fig. 11). Particular attention was paid during the implementation of the framework such that the work-flow of an intervention conducted on (quasi-)static anatomies remains unchanged, with the additional motion compensation features being hidden to the radiologist. Noteworthy is also the fact that the implementation and execution of the proposed framework was performed using commercially available hardware, all being integrated on an existing HIFU platform in conjunction with a standard 1.5 T Achieva MRI.

One of the drawbacks of the proposed motion correction framework is the lack of a component that addresses spontaneous motion. Thus, the latter is considered to be circumvented by some other means such as patient sedation. Problematic might also be the fact that the framework does not include an objective quality evaluation criterion for neither the MR images used for tracking, nor for the estimated displacements. While in the scope of this study, during the in-vivo experiments, a visual inspection of the MR images and the resulting estimated displacements was deemed sufficient, future studies need to address this issue by establishing an objective quality evaluation criterion. In addition, note that all deformations estimated and corrected by the proposed framework rely entirely on image registration algorithms. However, in particular for correcting geometric distortions, more specialized correction schemes can be employed. For example, the problem can be entirely avoided by an optimization of the acquisition parameters, such that it still allows thermometry with a high update rate, while at the same time minimizing geometric distortions. It is, however, difficult to predict whether such alternative approaches perform better than the proposed registration-based method, making this again the object of future studies.

## Conclusions

The present study proposes a motion correction framework encompassing the entire work-flow of an MRg-HIFU thermal therapy, ensuring spatial coherence between the different stages of the therapeutic work-flow. It was demonstrated that the framework allows both the adjustment of the interventional plan and projection of the therapy observables (e.g. temperature and thermal dose measurements) in a common frame-of-reference on-the-fly, with the patient on the interventional table. Furthermore, the proposed motion correction strategy was implemented as an additional feature directly into the clinical software stack, while at the same time maintaining compatibility with MRg-HIFU therapies for static anatomies.

## Abbreviations

CEM_43_: Cumulative equivalent minutes at 43°C
EPI: Echo planar imaging
EE: Endpoint error
HIFU: High intensity focused ultrasound
MRg-HIFU: Magnetic resonance-guided high intensity focused ultrasound
MRI: Magnetic resonance imaging
MIND: Modality independent neighborhood descriptor
PRFS: Proton resonance frequency shift
ROI: Region of interest
TR: Repetition time
TE: Echo time
